# Control of Aluminum and Titanium Contents in the Electroslag Remelting of ATI 718Plus^TM^ Alloy

**DOI:** 10.3390/ma17061254

**Published:** 2024-03-08

**Authors:** Lesong Li, Minqing Wang, Qintian Zhu, Xiaopeng Xu, Sifan Yu, Jian Zhang, Yang Zhou

**Affiliations:** 1 Central Iron and Steel Research Institute, Beijing 100081, China; 2 Gaona Aero Material Co., Ltd., Beijing 100081, China; 3 Chengdu Advanced Metal Materials Industry Technology Research Institute Co., Ltd., Chengdu 610000, China

**Keywords:** ATI 718Plus^TM^, electroslag remelting, thermodynamic model, ion and molecule coexistence theory

## Abstract

The burning loss of Al and Ti elements in superalloys during electroslag remelting has become a prevalent issue. And the existing slag system is not suitable for smelting the ATI 718Plus^TM^ alloy. Therefore, it is imperative to develop a new slag system for smelting the ATI 718Plus^TM^ alloy. To mitigate this issue, a thermodynamic model of the oxidation reaction of Al and Ti at the slag and alloy interface was established based on the ion and molecule coexistence theory (IMCT). The thermodynamic model was used to investigate the correlation between the equilibrium content of Al and Ti, slag composition, smelting temperature, and initial Al and Ti content of the electrode. The results indicate that while increasing the smelting temperature can effectively inhibit the burning loss of Al, it will exacerbate the burning loss of Ti. Increasing CaO and Al_2_O_3_ contents can inhibit the Al burning loss, while an increase in the TiO_2_ content can inhibit the Ti burning loss. Although an increase in the MgO content results in the burning loss of Al, its impact on the Al is minimal. The burning loss of Al and Ti was not affected by the change in the CaF_2_ content. The high Al content in ATI 718Plus^TM^ makes it prone to burning loss of Al during the electroslag remelting. The combustion loss of Al can be reduced by increasing the Ti content in the electrode or adding a suitable amount of aluminum powder to the slag system. The accuracy of the model had been validated through experimental verification.

## 1. Introduction

Superalloys refined by electroslag remelting have been widely used in aerospace, petrochemical, and other fields [1,2], so there are strict requirements on their properties. The principal components of the strengthening phase in superalloys are Al and Ti, and any alteration in the content of these elements within the alloy will have an impact on their high-temperature properties [3]. During the process of electroslag remelting, the Al and Ti elements react with unstable oxides, resulting in a loss of these elements due to burning, which has a significant impact on the quality of the electroslag ingot [4,5,6,7].

The primary approach to control the content of Al and Ti elements in superalloys during electroslag remelting is to minimize their interaction with the O element. The sources of the O element can be summarized as follows [8,9,10]: (1) the electroslag remelting is conducted in the ambient atmosphere, resulting in a reaction between oxygen and aluminum as well as titanium elements; (2) the electrode surface is not properly cleaned and contains impurities, such as FeO; (3) the slag system contains a small amount of SiO_2_; (4) the contents of Al_2_O_3_ and TiO_2_ in the slag are either excessive or insufficient. The primary factor among them is the irrational contents of Al_2_O_3_ and TiO_2_ in the slag. Therefore, it is imperative to investigate the impact of slag system constituents on the elements Al and Ti.

There have been reports regarding the control of Al and Ti in electroslag remelting. Pateishy [11] et al. studied the changes in Ti and Si contents during ESR and found that adding some TiO_2_ in slag could reduce the loss of Ti. Tan [12] et al. developed a thermodynamic model based on the ion and molecule coexistence theory, examining the impact of slag system components on the GH4065A alloy and identifying a slag system capable of inhibiting Al and Ti burning losses. Yang and Park [13] found that with increases in the reaction temperature, Ti was more easily oxidized than Al in nickel-based alloys. Therefore, more TiO_2_ should be added to the slag system at high temperatures. Jiang [14] et al. studied the relationship between the contents of Al, Ti, and Si in the GH8825 alloy and the content of CaO in the slag system and found that the higher the content of CaO in slag, the lower the loss of Ti via the reaction of Ti with the silica of the ESR slag. Duan [15] et al. systematically studied the effects of the slag composition and temperature on the contents of Al and Ti in the Inconel 718 alloy and found that the importance of factors for controlling the equilibrium Al content in the alloy was ordered as TiO_2_ > Al_2_O_3_ > CaO > CaF_2_ > MgO in ESR slags. In the research process, these scholars employed a methodology that integrates a thermodynamic model and experimental verification to derive effective measures for suppressing the combustion loss of Al and Ti elements.

Many scholars have established activity models suitable for different slag systems through experimental research, such as a regular solution model for molten slags [16], Temkin’s model [17], the ion and molecule coexistence theory model [18], and so on. The accuracy of the predictions made by many of these models is limited due to inherent limitations. The prerequisite for the application of the regular solution model for molten slag is to know the interaction energy between the cations of the components in the studied slag system. For a slag system with unknown interaction energy, it should be calculated based on test data [16]. The Temkin model only considers the most basic characteristics of slag, ignoring the difference in electrostatic potential when the ionic charge symbol is the same but the type and size are different, so the properties of the complete ionic solution are different from the actual slag [17]. The ion and molecule coexistence theory model obeys the law of mass action. Therefore, the known thermodynamic data, combined with the mass action law, can be used to predict the concentration of each structural unit in the slag system accurately [18]. Therefore, the ion and molecule coexistence theory model is utilized for constructing the thermodynamic model of the CaF_2_-CaO-MgO-Al_2_O_3_-TiO_2_ slag system.

The ATI 718Plus^TM^ alloy possesses a specific Al/Ti, setting it apart from other alloys. A slight modification in the composition of Al + Ti and Al/Ti has a significant impact on the type and quantity of precipitated phases as well as the mechanical properties of the alloy. The endurance life and thermal stability of the alloy increase significantly when n(Al + Ti) = 4at%, with an n(Al)/n(Ti) increase from 1 to 4. The maximum value is attained when the Al/Ti equals 4 [19]. Therefore, it is crucial to control the Al and Ti contents during the ESR of the ATI 718Plus^TM^ alloy. However, it is difficult to control the Al and Ti contents within a narrow range efficiently enough to meet the specific requirements for Al/Ti.

A thermodynamic model was developed based on the IMCT to describe the burning losses of Al and Ti elements in the ATI 718Plus^TM^ alloy during electroslag remelting. The influence of the slag system components, smelting temperature, and electrode element content on the Al and Ti contents of the ATI 718Plus^TM^ alloy were analyzed. The optimal slag system ratio for inhibiting the burning losses of Al and Ti elements has been determined. The model’s results have been validated through experimental verification.

## 2. Experimental

### 2.1. The Creation of a Thermodynamic Model

Firstly, the structural units of the slag system are determined by consulting the phase diagrams of the slag system. The relevant equations are then listed based on the principles of chemical equilibrium and mass conservation, followed by establishing a calculation model to determine the activity of each structural unit in the slag system. The activity of each structural unit of the slag system under different compositions and temperatures can be calculated by solving the equation with Matlab 2020a [20].

### 2.2. The Experiment on the Balance of Slag Alloy

**Figure 1:**
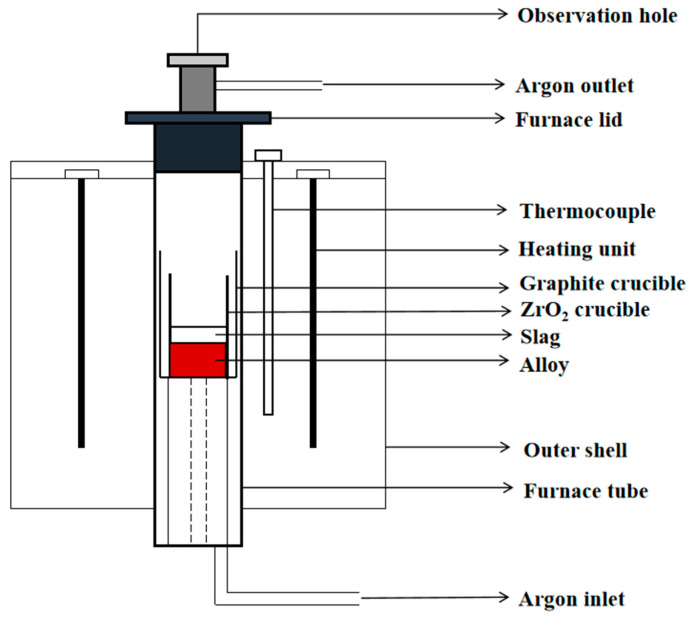
Schematic diagram of a resistance furnace.

The ATI 718Plus^TM^ alloy produced via vacuum induction melting was used in this experiment. The composition of the remelting slag was CaF_2_-CaO-MgO-Al_2_O_3_-TiO_2_. The schematic diagram of the experimental resistance furnace is depicted in Figure 1.

The first step involves placing 500 g of the alloy and 60 g of slag into a ZrO_2_ crucible, which is lined with a 0.2 mm thick molybdenum sheet. Then, the ZrO_2_ crucible should be placed inside the graphite crucible to prevent the liquid metal leakage under elevated temperature conditions. After placing the graphite crucible inside the resistance furnace, initiate the power supply of the resistance furnace and increase the temperature to 1873 K at a rate of 10 K/min. Maintain this temperature for a duration of 60 min, subsequent to which, turn off the power and permit the furnace to cool down to the ambient temperature. During the whole experiment, the resistance furnace is injected with argon gas at a flow rate of 5 L/min to prevent the reaction between oxygen and the liquid metal.

After the reaction between slag and the alloy, the electroslag ingot was axially sampled using the wire cutting method. Then, the Al and Ti element contents in the electroslag ingot were determined using inductively coupled plasma atomic emission spectrometry (ICP-AES).

## 3. Thermodynamic Model

During the electroslag remelting of the ATI 718Plus^TM^ alloy, when the electrode is in contact with the slag pool, the Al in the alloy reacts with the unstable TiO_2_ in the slag. The reaction is represented as Equation (1) [11].

4[Al] + 3[TiO_2_] ⇋ 3[Ti] + 2[Al_2_O_3_] (1)

(2) $${\log_{10}K}_{i}=\log_{10}\frac{a_{{\mathrm{Al}}_{2}O_{3}}^{2}*a_{\mathrm{Ti}}^{3}}{a_{{\mathrm{TiO}}_{2}}^{3}*a_{\mathrm{Al}}^{4}}=\log_{10}\frac{a_{{\mathrm{Al}}_{2}O_{3}}^{2}}{a_{{\mathrm{TiO}}_{2}}^{3}}+\log_{10}\frac{f_{\mathrm{Ti}}^{3}*{w([\mathrm{Ti}])}^{3}}{f_{\mathrm{Al}}^{4}*{w([\mathrm{Al}])}^{4}}=\frac{35,300}{T}-9.94$$

In the above Equation (2), K_i_ is the equilibrium constant for the reaction shown in Equation (1); $a_{{\mathrm{Al}}_{2}O_{3}}$ and $a_{{\mathrm{TiO}}_{2}}$ are the activities of Al_2_O_3_ and TiO_2_ in the slag; $a_{\mathrm{Al}}$ and $a_{\mathrm{Ti}}$ are the activities of Al and Ti; $f_{\mathrm{Al}}$ and $f_{\mathrm{Ti}}$ are the Henrian activity coefficients for Al and Ti; w_i_ is the mass fraction of element i in the metal; and T is the absolute temperature.

The activity of alloying elements is calculated as follows:

(3) $$\log_{10}f_{i}=\sum (e_{i}^{j}*w_{j})$$

(4) $$a_{i}=f_{i}*w_{i}$$

In the above Equation (3), $e_{i}^{j}$ is the activity coefficient of the alloying component element j on element i.

Table 1 shows the chemical composition of the ATI 718Plus^TM^ alloy used for developing the model. The S2059 slag system is selected as the base slag system, the composition of which is listed in Table 2. And Table 3 shows the activity interaction coefficient.

A phase diagram of the CaF_2_-CaO-MgO-Al_2_O_3_-TiO_2_ slag system reveals its 21 structural units, including four ions (Ca^2+^, Mg^2+^, F^2−^, and O^2−^), two simple molecules (Al_2_O_3_ and TiO_2_), and 15 complex molecules, such as 11CaO·7Al_2_O_3_·CaF_2._ Table 4 lists their molar number and mass action concentration, while Table 5 shows the chemical equations regarding the formation of complex molecules and their Gibbs free energies.

The chemical reaction of each component in the slag system follows the law of the conservation of mass. The molar fractions of each oxide in the slag system are denoted as b_1_ = n_CaF2_, b_2_ = n_CaO_, b_3_ = n_MgO_, b_4_ = n_Al2O3_, and b_5_ = n_TiO2_, and the total molar fraction is Σn_i_. The ratio of the amount of substance n_i_ of each structural unit in the slag system at reaction equilibrium to the total amount of substance Σn_i_ yields the mass action concentration N_i_ of each structural unit in the slag system. The mass action concentration refers to the concentration of a structural unit in a metallurgical melt that is subject to the law of mass action. The mass action concentration and activity are the same for pure matter as the standard state [28]. Therefore, as a description of the ion and molecule coexistence theory, the mass conservation equation was established in Equations (5)–(10); the nonlinear equations were then solved using MATLAB 2020a to obtain the mass action concentration of each structural unit.

(5) $$N_{1}+N_{2}+N_{3}+N_{4}+N_{5}+N_{c1}{+N}_{c2}+N_{c3}+N_{c4}+N_{c5}+N_{c6}+N_{c7}+N_{c8}+N_{c9}+N_{c10}+N_{c11}+N_{c12}+N_{c13}+N_{c14}+N_{c15}=\sum N_{i}=1$$

(6) $$b_{1}=\left(\frac{1}{3}N_{1}+N_{c14}+N_{c15}\right)\sum n_{i}=n_{{CaF}_{2}}$$

(7) $$b_{2}=\left(0.5N_{2}+N_{c1}+3N_{c3}+12N_{c4}+N_{c5}+N_{c6}+N_{c7}+3N_{c8}+4N_{c9}+3N_{c14}+11N_{c15}\right)\sum n_{i}=n_{CaO}$$

(8) $$b_{3}=\left(0.5N_{3}{+N}_{c2}+N_{c11}+N_{c12}+{2N}_{c13}\right)\sum n_{i}=n_{MgO}$$

(9) $$b_{4}=\left(N_{4}+N_{c1}{+N}_{c2}+N_{c3}+7N_{c4}+{2N}_{c5}+{6N}_{c6}+N_{c10}+3N_{c14}+{7N}_{c15}\right)\sum n_{i}=n_{{Al}_{2}O_{3}}$$

(10) $$b_{5}=\left(N_{5}+N_{c7}+2N_{c8}+3N_{c9}+N_{c10}+N_{c11}+2N_{c12}+N_{c13}\right)\sum n_{i}=n_{{TiO}_{2}}$$

The relation between the equilibrium contents of Al and Ti in the ATI 718Plus^TM^ alloy and the slag system components, smelting temperature, and initial alloying elemental contents is obtained from Equation (2); this relation is shown for Al and Ti as Equations (11) and (12), respectively.

(11) $${\mathrm{Log}}_{10}w\left(\left[\mathrm{Al}\right]\right)=\frac{1}{4}\left\{\log_{10}\frac{a_{{\mathrm{Al}}_{2}O_{3}}^{2}}{a_{{\mathrm{TiO}}_{2}}^{3}}-4\log_{10}f_{\mathrm{Al}}+3\log_{10}f_{\mathrm{Ti}}+3\log_{10}w\left(\left[\mathrm{Ti}\right]\right)-\left(\frac{35300}{T}-9.94\right)\right\}$$

(12) $$\log_{10}w\left(\left[\mathrm{Ti}\right]\right)=\frac{1}{3}\left\{\log_{10}\frac{a_{{\mathrm{TiO}}_{2}}^{3}}{a_{{\mathrm{Al}}_{2}O_{3}}^{2}}+4\log_{10}f_{\mathrm{Al}}-3\log_{10}f_{\mathrm{Ti}}+4\log_{10}w\left(\left[\mathrm{Al}\right]\right)+\left(\frac{35300}{T}-9.94\right)\right\}$$

The equilibrium Al and Ti contents are directly related to the activity of Al_2_O_3_ and TiO_2_, the smelting temperature, the initial Al and Ti contents of the alloy, and the activity in the slag. However, the equation does not depict the relationship between them and CaF_2_, CaO, and MgO in the slag. Therefore, it is necessary to analyze the influence of other components on the equilibrium content of Al and Ti elements in the slag system through theoretical calculations.

## 4. Results and Discussion

### 4.1. The Influence of Smelting Temperature on the Equilibrium Contents of Al and Ti

The temperatures at the electroslag remelting electrode, droplet, and molten bath are believed to be 1750 K, 1948 K, and 1948 K, respectively; the temperature of the slag–alloy interface is 1950 K, according to Mitchell [29]. Therefore, this paper selects five temperatures for research: 1773 K, 1823 K, 1873 K, 1923 K, and 1973 K.

**Figure 2:**
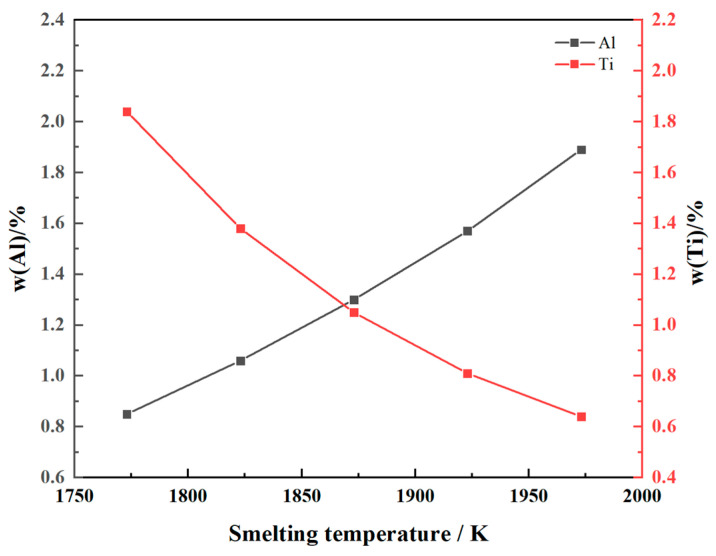
Relationship between equilibrium Al and Ti content and smelting temperature.

The influence of the smelting temperature on the equilibrium contents of Al and Ti when the composition of slag system components remains constant is illustrated in Figure 2. The figure demonstrates that an increase in the smelting temperature impedes the burning loss of Al, while a decrease in the smelting temperature hampers the burning loss of Ti. This phenomenon is consistent with the results of Shen et al. [30]. They point out that with temperature increases in a constant slag composition, the Ti content decreases drastically and the Al content increases.

The activity of Al_2_O_3_ and TiO_2_ increases with the rise in smelting temperature, while $lg\left(a_{{\mathrm{Al}}_{2}O_{3}}^{2}/a_{{TiO}_{2}}^{3}\right)$ exhibits a continuous decrease. This indicates that the content of TiO_2_ in the slag system increases after the chemical reaction. The reverse reaction of Equation (1) occurs at elevated temperatures, leading to an enhanced generation of Al elements and consequently an increase in the equilibrium Al content. A reduction in the smelting temperature, on the contrary, promotes the forward reaction in Equation (1) to enhance the production of Ti elements.

The minimum Al content of the ATI 718Plus^TM^ alloy is 1.2. According to the theoretical calculation results, when the melting temperature is lower than 1873 K, the burning loss of the Al element occurs easily. In order to ensure the properties of the alloy, the set temperature of electroslag remelting should not be lower than 1923 K.

### 4.2. The Influence of Components in the Slag System on the Equilibrium Contents of Al and Ti

**Figure 3:**
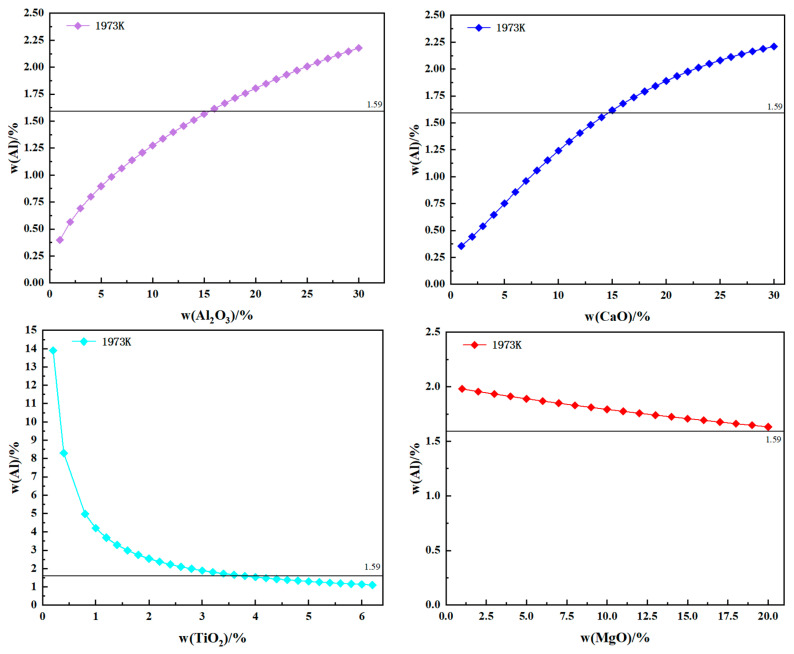

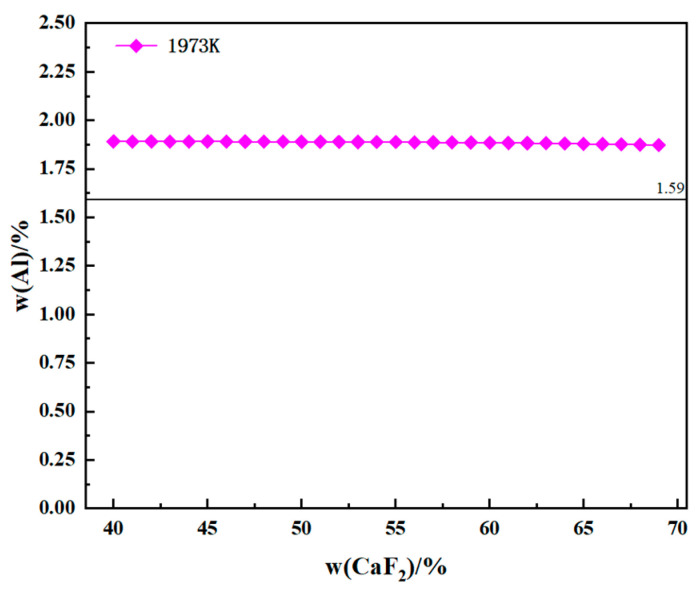
Relationship between equilibrium Al content and slag system component content.

**Figure 4:**
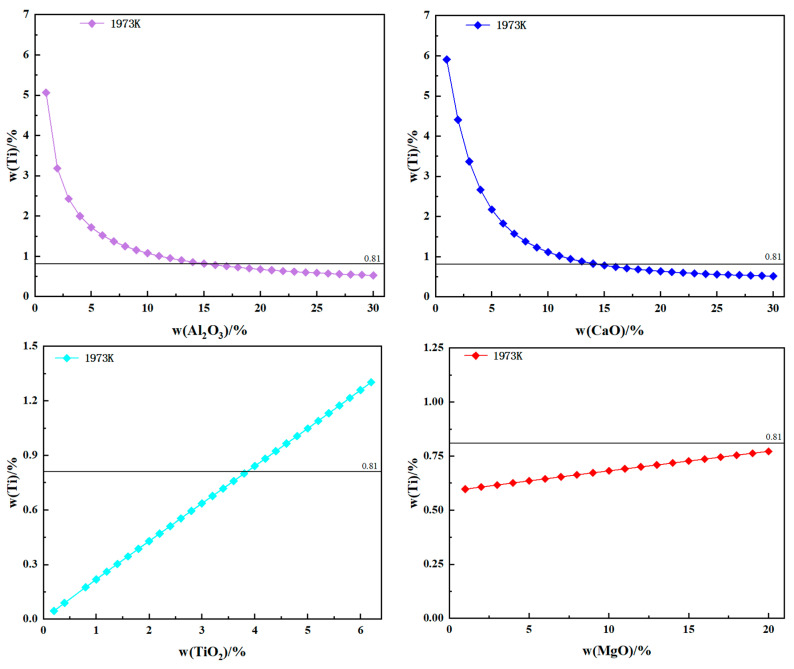

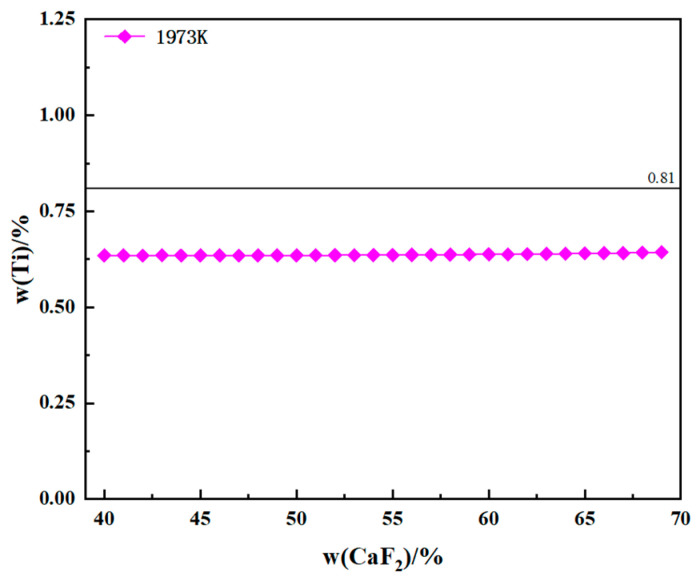
Relationship between equilibrium Ti content and slag system component content.

The relationship between the components of the slag system and the equilibrium contents of Al and Ti is investigated at a smelting temperature of 1973 K. The relationship between the content of Al and slag system components is illustrated in Figure 3. Similarly, Figure 4 illustrates the relationship between the content of Ti and slag system components. It is necessary to keep the proportional fractions of the contents of several other components in the slag constant when calculating the effect of one component of the slag system on the equilibrium Al and Ti contents. Take CaO as an example: when exploring the effect of CaO on the equilibrium Al and Ti content, the slag system group element mass fraction changes to CaF_2_:CaO:Al_2_O_3_:MgO:TiO_2_ = 50:X_CaO_:22:5:3, i.e., when the CaO mass fraction changes to 21%, the CaF_2_ mass fraction subsequently changes to 79 × 50/80 = 49.375%, and the other group element changes are also the same.

An increase in Al_2_O_3_ content, as depicted in Figure 3, leads to a significant rise in the equilibrium Al content. This can be attributed to the fact that Al_2_O_3_ consumes a substantial amount of Ti in the electrode, resulting in an increased generation of Al. Although excessive amounts of Al_2_O_3_ can increase the Al content in the electroslag ingot, it will result in the burning of Ti.

The content of the Al element increases with increases in the CaO content. This phenomenon can be attributed to the formation of complex molecules, such as CaO·TiO_2_, 3CaO·2TiO_2_, and 4CaO·3TiO_2_, due to the increased content of CaO. Consequently, the reaction between TiO_2_ and Al is reduced. However, when the CaO content is increased from 1% to 30%, the ratio of Al_2_O_3_ and TiO_2_ activities in the slag initially increases and then decreases. Moreover, when the mass fraction of CaO is 30%, the ratio of activities between Al_2_O_3_ and TiO_2_ is higher than that at a CaO mass fraction of 1%, indicating an increased degree of reaction between Ti and Al_2_O_3_, which results in an increase in the Al content in the electroslag ingot. Similarly, in Figure 4, the equilibrium Ti content decreases as the CaO and Al_2_O_3_ contents increase. This phenomenon is consistent with the results of Jiang et al. [14]. Their study concluded that the high CaO content in CaF_2_-CaO-Al_2_O_3_-MgO-TiO_2_-SiO_2_ slag can prevent the oxidation of Al in the GH8825 alloy.

The equilibrium Al content decreases with increases in the TiO_2_ content. However, the equilibrium Ti content increases with an increasing TiO_2_ content. This is because Equation (1) proceeds to the left of the reaction, increasing the Ti content by consuming more Al. However, an excessive TiO_2_ content will increase the Ti content and cause burning of Al.

As the MgO content increases, the equilibrium Al content decreases but remains higher than that of the initial electrode at a temperature of 1973 K. This is because Al reacts with MgO at high temperatures [31]: 2Al + 3MgO ⇋ 3Mg + Al_2_O_3_. However, the amount of the Al element consumed by MgO is little. Consequently, adjusting the MgO content allows for control over the Mg content in electroslag ingots. Similarly, increases in the MgO content result in a continuous rise in $\log_{10}\frac{a_{{\mathrm{TiO}}_{2}}^{3}}{a_{{\mathrm{Al}}_{2}O_{3}}^{2}}$, which promotes the forward reaction 4Al + 3TiO_2_ ⇋ 3Ti + 2Al_2_O_3_ and consequently enhances the equilibrium Ti content. The inhibitory effect of the MgO content on the combustion of Al and Ti is the same as that obtained by Duan [15] et al.

CaF_2_ has little effect on the equilibrium Al content and does not cause any burning loss of Al when changing the slag composition. And CaF_2_ has very little impact on Ti.

Thus, adjusting the contents of MgO and CaF_2_ can modify the physical properties of a slag system. And the Mg content in an electroslag ingot can be adjusted by adjusting the MgO content.

### 4.3. The Influence of the Initial Al and Ti Content in the Electrode on the Equilibrium Al and Ti Content of the Alloy

The content of Al and Ti elements in the initial electrode will also have an impact on the Al and Ti element contents of the electroslag ingot, in addition to factors such as slag system components and the smelting temperature. Therefore, the influence of the Al and Ti contents of the ATI 718Plus^TM^ alloy electrode on the equilibrium contents of Al and Ti in the electroslag ingot was studied.

**Figure 5:**
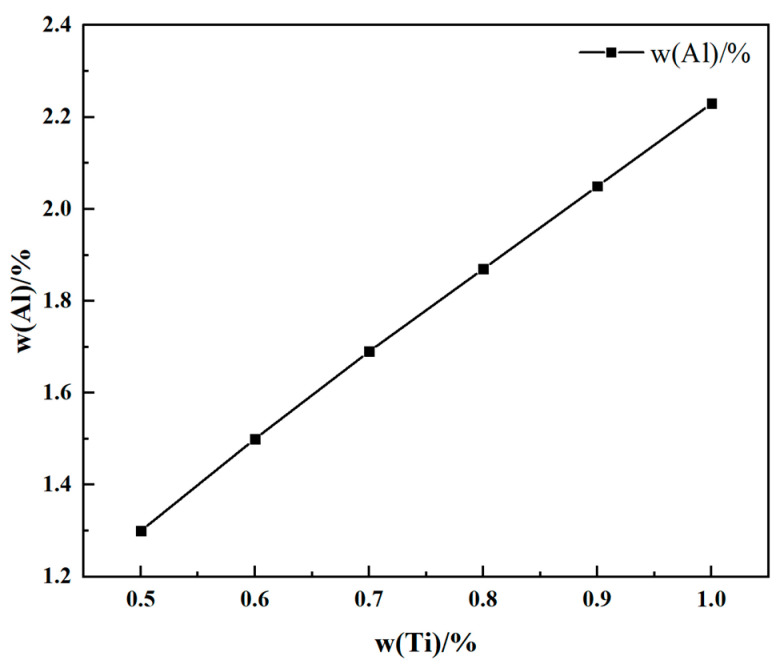
Relationship between equilibrium Al with different initial Ti contents.

**Figure 6:**
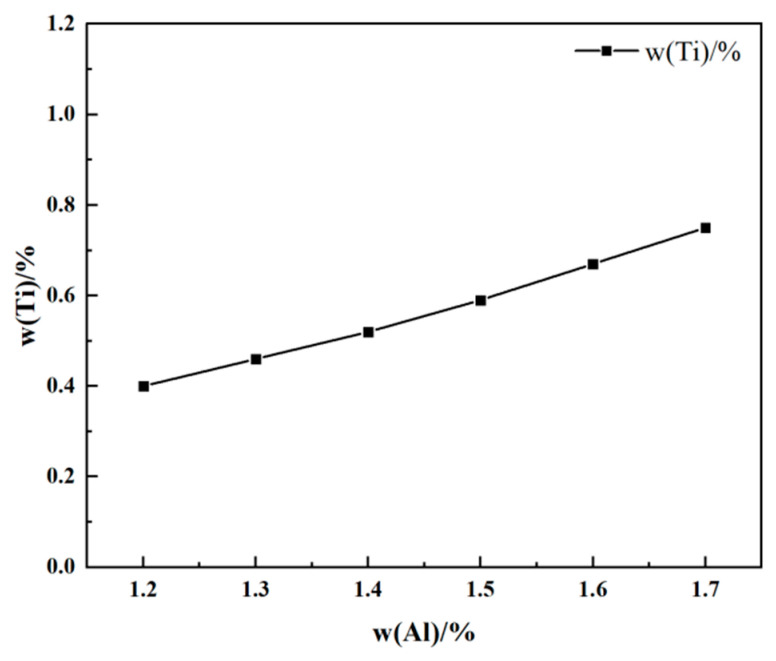
Relationship between equilibrium Ti with different initial Al contents.

Six different initial contents within the specified range of the Al and Ti elements of the ATI 718Plus^TM^ alloy were selected for this analysis at 1973 K. The effect of the slag system composition on the equilibrium Al content at varying initial Ti contents is shown in Figure 5. When the content of slag system components is constant, the equilibrium Al content increases with an increasing initial Ti content. This is because as the Ti content increases, its activity in the alloy strengthens, leading to a stronger reaction with Al_2_O_3_ in the slag and generating more Al contents. Thus, the burning loss of the Al element is inhibited. Similarly, as shown in Figure 6, the equilibrium Ti content increases with an increasing Al content in the alloy. The comparison of Figure 5 and Figure 6 reveals that the initial Ti content has a more significant influence on the equilibrium Al content of the alloy.

The ATI 718Plus^TM^ alloy is a high-Al and low-Ti superalloy that is more likely to produce the “burning Al increases Ti” phenomenon in the ESR. Therefore, for ensuring alloy performance stability and reducing the Al burning loss, either the Ti content in the electrode can be appropriately increased or an appropriate amount of Al powder can be added to the slag system.

### 4.4. Validation of Thermodynamic Model

**Figure 7:**
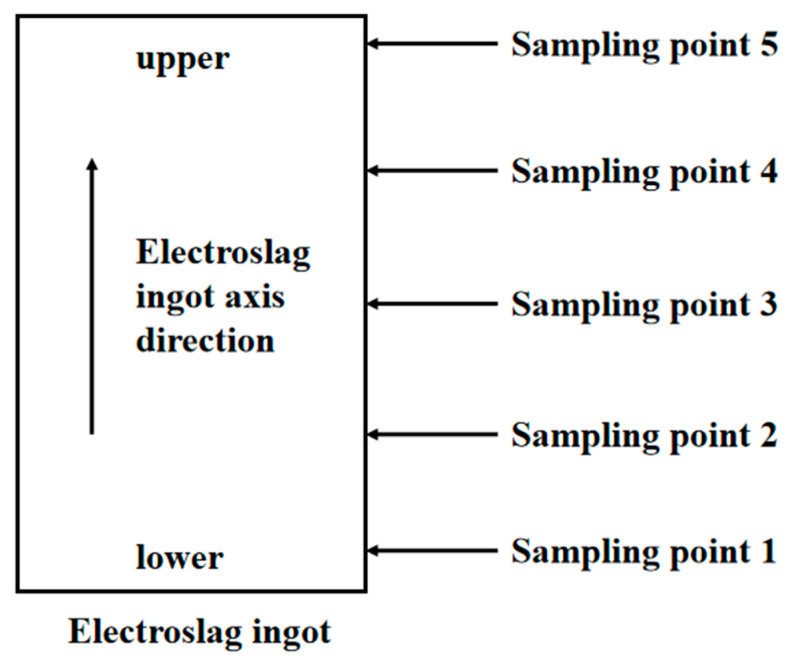
The sampling diagram of electroslag ingot.

Based on the theoretical calculations, the smelting temperature was elevated and adjustments were made to the content of each component in the slag system. The slag–alloy balance experiment was conducted using the adjusted slag system, and the smelting outcome was compared to that of the S2059 slag system. The electroslag ingot was sampled and analyzed after the experiment. The sampling diagram of the electroslag ingot is shown in Figure 7.

**Figure 8:**
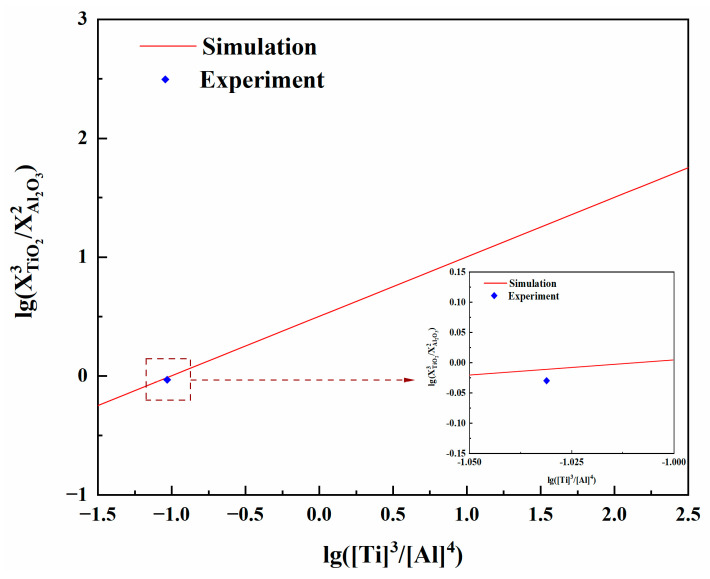
Dependence of the $\log_{10}(X_{{\mathrm{TiO}}_{2}}^{3}/X_{{\mathrm{Al}}_{2}O_{3}}^{2})$ on $\log_{10}({\left[Ti\right]}^{3}/{\left[Al\right]}^{4})$.

In order to verify whether the thermodynamic model is consistent with the experimental results, Equation (2) could be changed to Equation (13). The relationship between $\log_{10}(X_{{\mathrm{TiO}}_{2}}^{3}/X_{{\mathrm{Al}}_{2}O_{3}}^{2})$ and $\log_{10}({w\left[\mathrm{Ti}\right]}^{3}/{w[\mathrm{Al}]}^{4})$ can be obtained from the slag–alloy equilibrium test results and the interaction concentration of the slag system components calculated by the model. As shown in Figure 8, the experimental results are in good agreement with the model calculation, indicating that the established thermodynamic model is reasonable.

(13) $$\log_{10}\frac{X_{{\mathrm{TiO}}_{2}}^{3}}{X_{{\mathrm{Al}}_{2}O_{3}}^{2}}=\log_{10}\frac{{w\left[\mathrm{Ti}\right]}^{3}}{{w[\mathrm{Al}]}^{4}}+\log_{10}\frac{f_{\mathrm{Ti}}^{3}*\gamma_{{\mathrm{Al}}_{2}O_{3}}^{2}}{f_{\mathrm{Al}}^{4}*\gamma_{{\mathrm{TiO}}_{2}}^{3}}-\frac{35,300}{T}+9.94$$

(14) $$\gamma_{i}=\frac{N_{i}}{X_{i}}$$

In the above Equations (13) and (14), $\gamma_{{\mathrm{TiO}}_{2}}$ and $\gamma_{{\mathrm{Al}}_{2}O_{3}}$ express the activity coefficients of TiO_2_ and Al_2_O_3_ in slag and $X_{{\mathrm{TiO}}_{2}}$ and $X_{{\mathrm{Al}}_{2}O_{3}}$ express the mole fractions of TiO_2_ and Al_2_O_3_ in slag.

**Figure 9:**
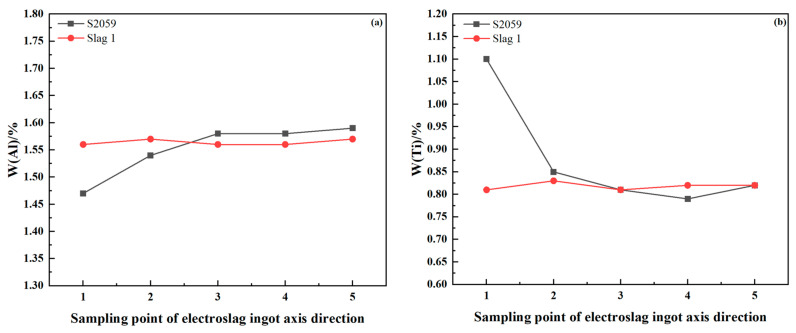
Distribution of elemental contents of Al and Ti in axial direction in electroslag ingot of ATI 718Plus^TM^ alloy: (**a**) w(Al)/%; (**b**) w(Ti)/%.

The distribution of Al and Ti elements in the electroslag ingot is illustrated in Figure 9 shows that the fluctuation ranges of Ti and Al contents are 0.01% and 0.02%, respectively, indicating that the precise control of Al and Ti contents has been preliminarily achieved in the ESR of the ATI 718Plus^TM^ alloy. Compared with the smelting results of the S2059 slag system, the distribution of Al and Ti elements along the axial direction of the electroslag ingot is uniform. In the beginning of smelting with the S2059 slag, the occurrence of “burning Al increases Ti” is observed. Due to the high TiO_2_ content in the S2059 slag system, a significant consumption of aluminum takes place during the initial smelting phase, resulting in an increased production of titanium. As smelting progresses and a portion of TiO_2_ in the slag becomes depleted, Equation (1) reaches a dynamic state.

## 5. Conclusions

Herein, the influence of the slag system components, smelting temperature, and electrode element content on the Al and Ti contents of the ATI 718Plus^TM^ alloy were analyzed by developing a thermodynamic model. The main conclusions obtained from the study are as follows:

1. Increases in the smelting temperature can effectively inhibit the burning loss of the Al element, while decreases in the smelting temperature can effectively inhibit the burning loss of the Ti element. In the electroslag remelting of the ATI 718Plus^TM^ alloy, it is necessary to appropriately increase the smelting temperature. The set temperature of electroslag remelting should not be lower than 1923 K.
2. Increasing the Al_2_O_3_ and CaO contents and decreasing the TiO_2_ and MgO contents in slag can inhibit the burning loss of Al; on the contrary, it can inhibit the burning loss of Ti. Varying CaF_2_ contents had little effect on the equilibrium content of Al and Ti contents; varying the MgO content could adjust the Mg content in the ATI 718Plus^TM^ alloy.
3. The initial Ti content of the electrode has a more significant influence on the equilibrium Al content of the alloy. Appropriately increasing the Ti content in the ATI 718Plus^TM^ alloy or adding Al powder to the slag can inhibit the burning loss of Al.

## Figures and Tables

**Table 1:** Chemical composition of ATI 718Plus^TM^ alloy (mass fraction).

Nb	Cr	Ni	Al	Ti	Mo
5.50	18.00	51.93	1.59	0.81	2.69

**Table 2:** Composition of base slag system (mass fraction).

Slag	CaF_2_	CaO	Al_2_O_3_	MgO	TiO_2_
S2059	50	20	22	5	3

**Table 3:** Activity interaction coefficients in nickel-based alloy melts in the present work [21,22,23].

$e_{j}^{i}$	Mn	Cr	Ni	Al	Ti	Mo
Al	0.034	0.045	−0.0376	0.0800	—	—
Ti	−0.12	0.025	−0.0166	—	0.0561	0.016

**Table 4:** Molar number and mass action concentration of structural units in 100 g CaF_2_-CaO-MgO-Al_2_O_3_-TiO_2_ slag system based on IMCT [24,25].

Items	Structural Unit	Serial Number	Moles of Structural Units n/mol	Mass Action Concentration of Structural Units N_i_
Ions	${Ca}^{2+}+{2F}^{2-}$	1	$n_{1}=n_{{Ca}^{2+},{CaF}_{2}}={2n}_{F^{-},{CaF}_{2}}$	$N_{1}=\frac{3n_{1}}{\sum n_{i}}N_{{CaF}_{2}}$
	${Ca}^{2+}+O^{2-}$	2	$n_{2}=n_{{Ca}^{2+},CaO}=n_{O^{2-},CaO}$	$N_{2}=\frac{2n_{2}}{\sum n_{i}}N_{CaO}$
	${Mg}^{2+}+O^{2-}$	3	$n_{3}=n_{{Mg}^{2+},MgO}=n_{O^{2-},MgO}$	$N_{3}=\frac{2n_{3}}{\sum n_{i}}N_{MgO}$
Molecules, composite molecules	${Al}_{2}O_{3}$	4	$n_{4}=n_{{Al}_{2}O_{3}}$	$N_{4}=\frac{n_{4}}{\sum n_{i}}N_{{Al}_{2}O_{3}}$
	${TiO}_{2}$	5	$n_{5}=n_{{TiO}_{2}}$	$N_{5}=\frac{n_{5}}{\sum n_{i}}N_{{TiO}_{2}}$
	$CaO\cdot {Al}_{2}O_{3}$	c1	$n_{c1}=n_{CaO\cdot {Al}_{2}O_{3}}$	$N_{c1}=\frac{n_{c1}}{\sum n_{i}}N_{CaO\cdot {Al}_{2}O_{3}}$
	$MgO\cdot {Al}_{2}O_{3}$	c2	$n_{c2}=n_{MgO\cdot {Al}_{2}O_{3}}$	$N_{c2}=\frac{n_{c2}}{\sum n_{i}}N_{MgO\cdot {Al}_{2}O_{3}}$
	$3CaO\cdot {Al}_{2}O_{3}$	c3	$n_{c3}=n_{3CaO\cdot {Al}_{2}O_{3}}$	$N_{c3}=\frac{n_{c3}}{\sum n_{i}}N_{3CaO\cdot {Al}_{2}O_{3}}$
	$12CaO\cdot {7Al}_{2}O_{3}$	c4	$n_{c4}=n_{12CaO\cdot {7Al}_{2}O_{3}}$	$N_{c4}=\frac{n_{c4}}{\sum n_{i}}N_{12CaO\cdot {7Al}_{2}O_{3}}$
	$CaO\cdot {2Al}_{2}O_{3}$	c5	$n_{c5}=n_{CaO\cdot {2Al}_{2}O_{3}}$	$N_{c5}=\frac{n_{c5}}{\sum n_{i}}N_{CaO\cdot {2Al}_{2}O_{3}}$
	$CaO\cdot {6Al}_{2}O_{3}$	c6	$n_{c6}=n_{CaO\cdot {6Al}_{2}O_{3}}$	$N_{c6}=\frac{n_{c6}}{\sum n_{i}}N_{CaO\cdot {6Al}_{2}O_{3}}$
	$CaO\cdot {TiO}_{2}$	c7	$n_{c7}=n_{CaO\cdot {TiO}_{2}}$	$N_{c7}=\frac{n_{c7}}{\sum n_{i}}N_{CaO\cdot {TiO}_{2}}$
	$3CaO\cdot {2TiO}_{2}$	c8	$n_{c8}=n_{3CaO\cdot {2TiO}_{2}}$	$N_{c8}=\frac{n_{c8}}{\sum n_{i}}N_{3CaO\cdot {2TiO}_{2}}$
	$4CaO\cdot {3TiO}_{2}$	c9	$n_{c9}=n_{4CaO\cdot {3TiO}_{2}}$	$N_{c9}=\frac{n_{c9}}{\sum n_{i}}N_{4CaO\cdot {3TiO}_{2}}$
	${Al}_{2}O_{3}\cdot {TiO}_{2}$	c10	$n_{c10}=n_{{Al}_{2}O_{3}\cdot {TiO}_{2}}$	$N_{c10}=\frac{n_{c10}}{\sum n_{i}}N_{{Al}_{2}O_{3}\cdot {TiO}_{2}}$
	$MgO\cdot {TiO}_{2}$	c11	$n_{c11}=n_{MgO\cdot {TiO}_{2}}$	$N_{c11}=\frac{n_{c11}}{\sum n_{i}}N_{MgO\cdot {TiO}_{2}}$
	$MgO\cdot 2{TiO}_{2}$	c12	$n_{c12}=n_{MgO\cdot 2{TiO}_{2}}$	$N_{c12}=\frac{n_{c12}}{\sum n_{i}}N_{MgO\cdot 2{TiO}_{2}}$
	$2MgO\cdot {TiO}_{2}$	c13	$n_{c13}=n_{2MgO\cdot {TiO}_{2}}$	$N_{c13}=\frac{n_{c13}}{\sum n_{i}}N_{2MgO\cdot {TiO}_{2}}$
	$3CaO\cdot 3{Al}_{2}O_{3}\cdot {CaF}_{2}$	c14	$n_{c14}=n_{3CaO\cdot 3{Al}_{2}O_{3}\cdot {CaF}_{2}}$	$N_{c14}=\frac{n_{c14}}{\sum n_{i}}N_{3CaO\cdot 3{Al}_{2}O_{3}\cdot {CaF}_{2}}$
	$11CaO\cdot 7{Al}_{2}O_{3}\cdot {CaF}_{2}$	c15	$n_{c15}=n_{11CaO\cdot 7{Al}_{2}O_{3}\cdot {CaF}_{2}}$	$N_{c15}=\frac{n_{c15}}{\sum n_{i}}N_{11CaO\cdot 7{Al}_{2}O_{3}\cdot {CaF}_{2}}$

**Table 5:** Chemical reaction formulae of possible composite molecules formed in 100 g CaF_2_-CaO-MgO-Al_2_O_3_-TiO_2_ five-membered slag system [26,27].

Chemical Equation	$∆G_{i}^{\theta }/\left(J\cdot {mol}^{-1}\right)$	$N_{i}$
$\left({Ca}^{2+}+O^{2-}\right)+\left({Al}_{2}O_{3}\right)=\left(CaO\cdot {Al}_{2}O_{3}\right)$	59413−59.413T	$N_{c1}=K_{c1}N_{2}N_{4}$
$\left({Mg}^{2+}+O^{2-}\right)+\left({Al}_{2}O_{3}\right)=\left(MgO\cdot {Al}_{2}O_{3}\right)$	−18828−6.276T	$N_{c2}=K_{c2}N_{3}N_{4}$
$3\left({Ca}^{2+}+O^{2-}\right)+\left({Al}_{2}O_{3}\right)=\left(3CaO\cdot {Al}_{2}O_{3}\right)$	−21757−29.288T	$N_{c3}=K_{c3}N_{2}^{3}N_{4}$
$12\left({Ca}^{2+}+O^{2-}\right)+7\left({Al}_{2}O_{3}\right)=\left(12CaO\cdot {7Al}_{2}O_{3}\right)$	617977−612.119T	$N_{c4}=K_{c4}N_{2}^{12}N_{4}^{7}$
$\left({Ca}^{2+}+O^{2-}\right)+2\left({Al}_{2}O_{3}\right)=\left(CaO\cdot 2{Al}_{2}O_{3}\right)$	−16736−25.522T	$N_{c5}=K_{c5}N_{2}N_{4}^{2}$
$\left({Ca}^{2+}+O^{2-}\right)+6\left({Al}_{2}O_{3}\right)=\left(CaO\cdot 6{Al}_{2}O_{3}\right)$	−22594−31.798T	$N_{c6}=K_{c6}N_{2}N_{4}^{6}$
$\left({Ca}^{2+}+O^{2-}\right)+\left({TiO}_{2}\right)=\left(CaO\cdot {TiO}_{2}\right)$	−79900−3.35T	$N_{c7}=K_{c7}N_{2}N_{5}$
$3\left({Ca}^{2+}+O^{2-}\right)+2\left({TiO}_{2}\right)=\left(3CaO\cdot {2TiO}_{2}\right)$	−207100−11.35T	$N_{c8}=K_{c8}N_{2}^{3}N_{5}^{2}$
$4\left({Ca}^{2+}+O^{2-}\right)+3\left({TiO}_{2}\right)=\left(4CaO\cdot {3TiO}_{2}\right)$	−292880−17.573T	$N_{c9}=K_{c9}N_{2}^{4}N_{5}^{3}$
$\left({Al}_{2}O_{3}\right)+\left({TiO}_{2}\right)=\left({Al}_{2}O_{3}\cdot {TiO}_{2}\right)$	−25270+3.924T	$N_{c10}=K_{c10}N_{4}N_{5}$
$\left({Mg}^{2+}+O^{2-}\right)+\left({TiO}_{2}\right)=\left(MgO\cdot {TiO}_{2}\right)$	−26400+3.14T	$N_{c11}=K_{c11}N_{3}N_{5}$
$\left({Mg}^{2+}+O^{2-}\right)+2\left({TiO}_{2}\right)=\left(MgO\cdot 2{TiO}_{2}\right)$	−27600+0.63T	$N_{c12}=K_{c12}N_{5}^{2}N_{3}$
$2\left({Mg}^{2+}+O^{2-}\right)+\left({TiO}_{2}\right)=\left(2MgO\cdot {TiO}_{2}\right)$	−25500+1.26T	$N_{c13}=K_{c13}N_{5}N_{3}^{2}$
$3\left({Ca}^{2+}+O^{2-}\right)+3\left({Al}_{2}O_{3}\right)+\left({Ca}^{2+}+2F^{-}\right)=\left(3CaO\cdot {3Al}_{2}O_{3}\cdot {CaF}_{2}\right)$	−44492−73.15T	$N_{c14}=K_{c14}N_{1}{N_{2}^{3}N}_{4}^{3}$
$11\left({Ca}^{2+}+O^{2-}\right)+7\left({Al}_{2}O_{3}\right)+\left({Ca}^{2+}+2F^{-}\right)=\left(11CaO\cdot {7Al}_{2}O_{3}\cdot {CaF}_{2}\right)$	−228760−155.8T	$N_{c15}=K_{c15}N_{1}{N_{2}^{11}N}_{4}^{7}$

## Data Availability

Data are contained within the article.
